# Standard Precautions: Occupational Exposure and Behavior of Health Care Workers in Ethiopia

**DOI:** 10.1371/journal.pone.0014420

**Published:** 2010-12-23

**Authors:** Ayalu A. Reda, Shiferaw Fisseha, Bezatu Mengistie, Jean-Michel Vandeweerd

**Affiliations:** 1 Department of Public Health, College of Health Sciences, Haramaya University, Harar, Ethiopia; 2 Environmental Health Section, East Hararghe Zone Health Bureau, Harar, Ethiopia; 3 Department of Environmental Health Science, College of Health Sciences, Haramaya University, Harar, Ethiopia; 4 University of Namur, FUNDP, Namur, Belgium; National AIDS Research Institute, India

## Abstract

**Background:**

Occupational exposure to blood and body fluids is a serious concern for health care workers, and presents a major risk for the transmission of infections such as HIV and hepatitis viruses. The objective of this study was to investigate occupational exposures and behavior of health care workers (HCWs) in eastern Ethiopia.

**Methods:**

We surveyed 475 HCWs working in 10 hospitals and 20 health centers in eastern Ethiopia using a structured questionnaire with a response rate of 84.4%. Descriptive statistics and multivariate analysis using logistic regression were performed.

**Results:**

Life time risks of needle stick (30.5%; 95% CI 26.4–34.6%) and sharps injuries (25.7%; 95% CI 21.8–29.6%) were high. The one year prevalence of needle stick and sharps injury were 17.5% (95% CI 14.1–20.9%) and 13.5% (95% CI 10.4–16.6%) respectively. There was a high prevalence of life time (28.8%; 95% CI = 24.7–32.9%) and one year (20.2%; 95% CI = 16.6–23.8%) exposures to blood and body fluids. Two hundred thirteen (44.8%) HCWs reported that they were dissatisfied by the supply of infection prevention materials. HCWs had sub-optimal practices and unfavorable attitudes related to standard precautions such as needle recapping (46.9%) and discriminatory attitudes (30.5%) toward HIV/AIDS patients.

**Conclusion:**

There was a high level of exposure to blood and body fluids among HCWs. We detected suboptimal practices and behavior that put both patients and HCWs at significant risk of acquiring occupational infections. Health authorities in the study area need to improve the training of HCWs and provision of infection prevention equipment. In addition, regular reporting and assessment of occupational exposures need to be implemented.

## Introduction

Occupational exposure to blood and body fluids is a serious concern for health care workers and presents a major risk for the transmission of infections such as human immuno-deficiency virus (HIV), hepatitis B virus (HBV), and hepatitis C virus (HCV) [1], [2], [3]. Recognizing this threat, the U.S. Centers for Disease Control and Prevention (CDC) proposed a series of procedures for preventing occupational exposures and for handling potentially infectious materials such as blood and body fluids. These procedures, known as standard precautions (SPs), advise health care workers (HCWs) to practice regular personal hygiene; use protective barriers such as gloves and gown whenever there is contact with mucous membranes, blood and body fluids of patients; and dispose of sharps, body fluids, and other clinical wastes properly [4], [5], [6].

The World Health Organization (WHO) estimates that about 3 million HCWs face occupational exposure to blood borne viruses each year (2 million to HBV, 900,000 to HCV, and 300,000 to HIV), 90% of the infections that result from these exposures are in low income countries [2], [7]. Developing countries, especially those in sub-Saharan Africa, that account for the highest prevalence of HIV-infected patients in the world also report the highest incidences of occupational exposures [2], [3], [8].

Reports indicate that SPs are effective in preventing both occupational exposure incidents and associated infections [3], [9]. Due to this, surveillance of HCWs' compliance to SPs is an important element of occupational and nosocomial infection control as it enables assessment of risks from occupational exposure to infection. Studies have extensively reported suboptimal and non-uniform adherence to SPs by HCWs in both developed and developing countries [2], [10]–[12]. For instance only 58% of nurses from a study in Australia reported using gloves when handling ‘blood or blood equipment’ [13]. Up to half of HCWs from southern Ethiopia recapped needles [14], a third of HCWs from a study in Nigeria reported to always recap [15], while 40% from a study in India recapped at least sometimes and only 32% wore eye protection when indicated [16].

HCV and HBV infections are generally considered endemic in sub-Saharan Africa [8]. National data are unavailable for these blood borne infections in Ethiopia. However, surveys in different parts of the country indicate the prevalence of HCV to be 0.9–5.8% [17], [18] and estimates for HBV range from 4.7% to 14.4% [18]–[22]. According to projections for 2010, the prevalence of HIV/AIDS for Ethiopia is estimated at 2.8% [23]. Blood is routinely screened before transfusion. An official supply of disposable syringes and related devices are available even though little is known about their adequacy and replenishment.

In Ethiopia, there are only a few studies that describe occupational exposures and compliance to SPs among HCWs. In 2006, the Ethiopian Public Health Association indentified standard precautions as an area of research gap and public health importance in the country citing lack of investigations in this area and the apprehension of HCWs in handling HIV/AIDS cases [24]. Since then, the governmental and non-governmental organizations (NGOs) have given attention to standard precautions by initiating post-exposure prophylaxis (PEP) and increased supply of materials such as safety boxes. However, the evidence base surrounding SPs in this resource poor setting remains limited. This study aims to investigate occupational exposure and the behaviour of health care workers in eastern Ethiopia.

## Methods

### Settings, study design and participants

We conducted a cross-sectional survey in 10 hospitals and 20 health centers in two administrative regions of Ethiopia (Harari and Dire Dawa). All health care personnel including physicians, nurses, laboratory technicians and health assistants, working in the institutions and directly involved in day-to-day patient care were included in the study. The researchers reached participants through their respective institution and department heads. Data collection took place from February 1 to May 30, 2010.

Ethiopian health care institutions are structured according to the World Health Organization's recommendation for primary health care [25] and consist of community health centers and hospitals with governmental and private ownership. The surveyed institutions serve more than 620,000 people residing in urban and rural areas [26]. Projected estimates of HIV/AIDS prevalence for 2010 are 4.4% for Harari and 5.7% for Dire Dawa [23]. There is limited information on the prevalence of blood borne infections in the study area apart from HIV/AIDS. In addition, limited information is available on routes of transmission such as traditional practices and injection drug use.

### Questionnaire and data collection

Data were collected using a self administered structured questionnaire. It was developed after reviewing qualitative and quantitative literatures for relevant items. Final items were generated after running a partial Delphi process. This is an interactive and multistage group facilitation technique designed to transform opinion into group consensus [27]. After consensus, the items were checked for clarity and translated into the local language of Amharic. The resulting questionnaire was pretested on a convenience sample of 30 HCWs in a nearby health center in another neighboring region (Oromia) and corrections were made afterwards. The final questionnaire with 58 close ended questions included basic demographic information such as age and sex; behavior and attitudes toward standard precautions; and occupational exposure incidents. The questionnaire specifically asks respondents to list life time and previous one year exposures specifically from needle-stick injuries, other sharps injuries, and blood and body fluids splashing (refer to supporting File S1 for questionnaire).

### Statistical analysis

SPSS version 15.0 was used for data analysis. Associations were examined using Chi-square tests and multivariate logistic regression. Multicollinearity was examined using linear regression. Unadjusted and adjusted (AOR) odds ratios were used as indicators of the strength of association. Alpha was set at less than or equal to 0.05.

### Ethical clearance

The Institutional Research Ethics Review Committee of Haramaya University gave ethical approval. The HCWs were informed about the study, its importance and confidentiality of the information requested. Written consent was obtained from participants in a form provided with the questionnaire.

## Results

### Population

From among a total of 563 HCWs working in 30 health care institutions in the area, 484 responded giving a response rate of 84.4%. We discarded 9 incomplete questionnaires, and based our analysis on the remaining 475 respondents. The mean age and work experience of the respondents were 30.8 (SD±8.9) and 8.2 years (SD±8.7) respectively (Table 1).

**Table 1:** Characteristics of the study population (N = 475).

Characteristic	n (%)
Male sex	244 (53.4%)
Age, mean (SD)	30.8 (8.9)
Work experience, mean (SD)	8.2 (8.7)
Profession	
Nurses	333 (70.1)
Laboratory Technicians	47 (9.9)
Health officers	32 (6.7)
Midwives	28 (5.9)
Health Assistants	20 (4.2)
Physicians	15 (3.2)
Health care institution	
Hospitals	10 (33.3)
Health centers	20 (66.7)
Employment	
Hospital	301 (63.4)
Health center	174 (36.6)

### Occupational exposure

The self-reported life time risk of at least one needle stick or sharps injury among HCWs was 30.5% (95% CI 26.4–34.6%), and 25.7% (95% CI 21.8–29.6%) respectively. The self-reported one year prevalence of needle stick- and sharps injury was 17.5% (95% CI 14.1–20.9%) and 13.5% (95% CI 10.4–16.6%) respectively. The self-reported life time- and one year risk of splashing of blood and body fluids was 28.8% (95% CI 24.7–32.9%) and 20.2% (95% CI 16.6–23.8%) (Table 2).

**Table 2:** Responses of HCWs to items related to standard precautions (N = 475).

Items		% of ‘yes’ or ‘agree’ responses (n)
**I**	**Self-reported life time and last one year exposure incidences**	
	Have you ever had needle stick injury?	30.5% (145)
	Have you had needle stick injury in the last one year?	17.5% (83)
	Have you ever had sharps injury?	25.7% (122)
	Have you had sharps injury in the last one year?	13.5% (64)
	What were the reasons for the last needle stick injury in the last one year?	
	Sudden movement of the patient	45% (36)
	During recapping	36.3% (29)
	During handling and collection of wastes	18.8% (15)
	Have you ever had splashing of blood or body fluids to your mouth or eyes?	28.8% (137)
	Have you had splashing of blood or body fluids to your mouth or eyes in the last one year?	20.2% (96)
	What measures did you take after exposure to blood or body fluids or injury with needle stick or sharps?	
	Washing with soap and water	43.4% (206)
	Wash with iodine or alcohol solution	38.5% (183)
	Get tested for HIV	26.5% (126)
	Seek post exposure prophylaxis (PEP)	18.5% (88)
	Take tetanus anti-toxoid (TAT)	10.7% (51)
	Squeezing to extract more blood	10.1% (48)
	Applying pressure to stop bleeding	9.1% (43)
**II**	**Self-reported behavior, knowledge and attitude**	
	Which of the following infections are transmitted through blood and body fluids?	
	Human immunodeficiency virus (HIV)	97.9% (465)
	Hepatitis B virus (HBV)	91.8% (436)
	Hepatitis C virus (HCV)	61.7% (293)
	Malaria	15.6% (74)
	Which of the factors below do you think are contributing to occupational exposures in your institution	
	Lack of personal protection equipment (PEP)	70.1% (333)
	Inadequate hand washing facility	65.7% (312)
	Excess work load	64.6% (307)
	Over crowded work place	61.1% (290)
	Lack of commitment to invest in infection control by management	60.4% (287)
	Is there a tendency to over-prescribe injections in your health institution?	23.4% (111)
	Have you worn gloves the last time you took a blood sample?	82.5% (392)
	Have you taken training on occupational infection prevention?	39.6% (188)

Working in hospitals was associated with risk of needle stick injury (OR 3.2; 95% CI 2.2–4.8; AOR 1.75; 95% CI 0.96–1.10), sharps injury (OR 2.2; 95% CI 1.2–4.0) but not with body fluids splashing to mouth or eyes (OR 1.7; 95% CI 1.05–2.8; AOR 1.53; 95% CI 0.89–2.62) in the past one year. Needle stick injury was significantly associated with females (AOR 1.75; 95% CI 1.04–2.92) and HCWs that practice needle recapping, but it failed to reach statistical significance (AOR 1.27; 95% CI 0.76–2.13). Findings of the multivariate logistic regression analysis are displayed in Table 3. Taking training was not protective against needle stick injury in the past one year (OR 0.9; 95% CI 0.6–1.5). Needles stick injury (p 0.53) or body fluid splashing to the eyes and mouth (p.06) were not significantly different across professions. The main reasons for the last injury in the past one year were sudden movement of the patient (45%) and recapping (36.3%). Last one year incidence of needle stick injury and blood and body fluids splashing were significantly associated with each other independently (OR 3.20; 95% CI 1.91–5.37; AOR 3.21; 95% CI 1.86–5.48). The measures HCWs took in the event of occupational exposures and injuries included PEP (88, 18.5%) and getting tested for HIV (126, 26.5%) (Table 2).

**Table 3:** Multivariate logistic regression model (1 and 2) results.

Model 1. Risk of needle stick injury in the past one year (self-reported)§			
Variables	OR	95% CI	P
Age	1.03	0.96–1.10	0.38
Sex (Female vs. Male)	1.75	1.04–2.92	0.03*
Institution (Hospital vs. Health centre)	1.75	0.96–3.19	0.06
Experience	0.99	0.92–1.05	0.73
Regularly apply standard precautions¥	1.09	0.88–1.35	0.40
Recap needles (Yes vs. No)	1.27	0.76–2.13	0.36
Blood and body fluids splashing to eyes or mouth in the past one year	3.21	1.88–5.48	<0.00**

Model 2. Risk of blood and body fluids splashing to the eye or mouth in the past one year(self-reported)§			
Age	0.98	0.91–1.05	0.48
Sex (Female vs. Male)	1.002	0.62–1.62	0.99
Institution (Hospital vs. Health centre)	1.53	0.89–2.62	0.12
Experience	1.03	0.96–1.10	0.44
Regularly apply standard precautions¥	0.79	0.66–0.96	0.02*
Recap needles (Yes vs. No)	0.96	0.59–1.57	0.88
Needles stick injury in the past one year (Yes vs. No)	3.17	1.86–5.42	<0.00**

¥ Rated on a Likert scale from 1 ‘Never’ to 5 ‘Always’;

§ -2LL = 449.3; Nagelkerke R Square = 9.0%; Model p<0.00;

§ -2LL = 404.0; Nagelkerke R Square = 10. 9%; Model p<0.00;

*p<0.05;

**p<0.01; OR, odds ratio; CI, confidence interval.

### Behavior and attitudes

Two hundred thirteen (44.8%) HCWs reported that they were dissatisfied by the supply of infection prevention materials, while three hundred thirty seven (70.9%) respondents perceived their work place to have put them at high risk of HIV. One hundred forty five (30.5) HCWs reported that HIV patients should be cared for separate from other patients. One hundred forty four (30.3%) participants reported that patients may have acquired nosocomial HIV infection. This response was significantly associated with HCWs working in hospitals (OR 2.2; 95% CI 1.4–3.4).

Three hundred eighty four (80.8%) HCWs reported that they regularly follow standard precautions. Two hundred thirty three (46.9%) HCWs practice needle recapping, while 28 (5.9%) reuse syringes, the main reason cited for reuse being shortage (78.5%, n 22). Those working at hospitals had a higher frequency of needle re-use (OR = 2.8; 95% CI 1.04–7.5) and recapping (OR 3.2; 95% CI 2.2–4.8) compared to their peers in health centers. Recapping was also highest among physicians (73.3%), health assistants (65.0%) and laboratory technicians (57.4%) (p 0.04). Three hundred seventy nine (79.8%) HCWs responded that gloves are required for any contact with patients (Table 2).

## Discussion

We detected a high level of self-reported exposure to blood and body fluids. Life time risks of needle stick (30.5%) and sharps injuries (25.7%) were high. There was a high prevalence of both life time (28.8%) and one year (20.2%) exposures to body fluids. HCWs had poor practices like needle recapping (46.9%) and unfavorable attitudes such as discriminatory opinions (30.5%) toward HIV/AIDS patients.

The logistic regression model indicated that needle stick injuries and body fluid exposures are strongly associated with each other, indicating the clustering nature of exposure incidents on groups of HCWs probably based on risky habits and suboptimal SPs compliance. The 17.5% one year prevalence of needle stick injury is similar to a report from the United Arab Emirates (UAE) by Jacob et al. [28] in which 19% of HCWs faced injury, but lower than a finding in northern Ethiopia [29] in which a three month prevalence of 17.2% was reported; and much lower than that of a report from southern Ethiopia in which the one year prevalence was 30.9% [14] and a study in Uganda (57%) by Nsubuga et al. [30]. A higher percentage of respondents (47%) in this study as well as in southern Ethiopia [14] (57%) had risky practices such as needle recapping. The risks of infection following percutaneous exposure to infected blood is lower for HIV (0.3%) [31], [32] compared to hepatitis C (3%) and hepatitis B (30%) [32]. However, this is not reassuring as the higher frequency of injury and exposures reported and the high prevalence of these infections mean that HCWs in developing countries are at a magnified risk of acquiring the infections.

The level of training about SPs by the current participants (39.6%) is similar to a finding in India by Kermode et al. [16] in which 36% HCWs have taken training. Unfortunately, taking training was not found to be protective from occupational exposures such as needle stick injury in the past one year (OR 0.9; 95% CI = 0.6–1.5). This will be a serious challenge to infection prevention efforts. This is similar to previous reports [14], [33], [34] in which training to HCWs seems to not necessarily bring about protection from exposures. The reason for this may be that the knowledge acquired may not necessarily translate into practice of preventive measures or that the trainings provided may be more theoretical than practical and the limited sources of continuous information on standard precautions. Lack of an enabling environment to comply with standard precautions may also contribute to poor compliance.

In this study 80.8% of HCWs reported that they regularly follow SPs and the regression model indicated that HCWs who regularly apply standard precautions reduced their risks to exposure incidents by 20%. The level of compliance in this study similar is to a finding from Australia [13] and higher than the report from the UAE (19%) [28]. However, when we consider contradictory findings such as belief by 79.8% HCWs that gloves are required for any contact with patients, and 46.6% recapped needles, we know that the rate of proper compliance is probably much lower, as documented in a study in Australia [13]. In addition, HCWs commonly over estimate their knowledge and practices on infection prevention [13], [16], [35], the magnitude of which is methodologically difficult to estimate. Partial compliance and suboptimal practices were also reported in other countries such as Nigeria [15], India [16] and the UK [36] where HCWs make unjustified assessments of risks from- and infection status of clients rather than properly and consistently applying standard precautions.

About 45% of the participants reported dissatisfaction by the provision of infection prevention materials (44.8%); and reuse of syringes was practiced by 5.9% HCWs, 78.5% of whom cited lack of supply as the main reason. Lack of infection prevention supplies seriously hampers prevention efforts and puts patients and HCWs at greater risk of infection and adds to the dissatisfaction of HCWs with their work environment. In fact, on top of the dangerous practice of needle reuse in a minority of HCWs, the report by 30.3% that patients may have acquired nosocomial HIV infection is a worrisome finding that adds credence to the foregoing argument. It is known that HCWs in sub-Saharan Africa are dissatisfied with their job, underpaid and overworked, and ill-protected [8], [37]–[39]. Even though the authors of this report have witnessed an increased attention to provision of infection prevention materials recently, such findings indicate the need to further increase supplies. Furthermore, 70.9% of the HCWs perceived their work place to have put them at higher risk of acquiring HIV infection and 30.5% preferred treating HIV patients separate from other patients. This may indicate the general level of apprehension in the work environment and the associated stigma toward HIV/AIDS patients on the part of HCWs in this and previous surveys in the country [24], [34] and elsewhere [40]–[42].

There have been very few randomized controlled trials conducted that provide evaluative evidence for effectiveness of interventions for reducing occupational exposure [31]. The commonly recommended preventive strategies for reducing occupational injuries and to increase conformity with standard precautions include education, awareness campaigns, risk reducing devises such as single use needles, reduction of unnecessary injections, legislative action, provision of personal protective equipment (PPE), introduction of safety guidelines and reporting mechanisms, and creating a compliance-enabling environment [3], [32], [43], [44]. Involvement of HCWs in infection control decisions is considered important [31]. Efforts toward reducing population levels of infections such as hepatitis and HIV are also important goals. However, it is known that these preventive strategies are mostly not implemented fully and/or compromised in the health care systems of most developing countries [3], [8], [28], [30], [44]. In Ethiopia, despite recognition of the importance of HIV/AIDS and other diseases transmitted through blood and body fluids by policy makers and public health professionals alike [24], there are no formalized post-exposure counseling, reporting procedures and infection control strategies in general. For instance, hepatitis and tetanus vaccinations remain inaccessible for Ethiopian HCWs. In addition, the recently launched post exposure prophylaxis (PEP) against HIV infection is available only at selected hospitals in urban areas and as a result it is not easily accessible to most HCWs. Therefore, governmental and non-governmental organizations need to expand and revise the currently available prevention facilities and put in place infection control and prevention strategies that are locally sustainable, cost-effective and scientifically sound.

The response rate of 84.4% is higher [13], [28], [36] or similar [16] to previous studies. No particular characteristic could be identified in non-respondents except that some HCW were unavailable as they had either joined short courses or enrolled in higher institutes for further study. Social desirability bias is also a potential limitation in self-reported studies like this one, in that HCWs might report socially acceptable responses than their actual day to day practice. The lack of standardized questionnaires with acceptable reliability and validity for assessing compliance to standard precautions limits comparison of our findings with previous research. To overcome this problem we included items used by other authors in order to aid comparison. As this is a cross-sectional study, the limitations that come with this type of design need to be taken into consideration when interpreting the findings.

We conclude that there is a high level of exposure to blood and body fluids among health care workers in the study area. We detected suboptimal practices and behavior that put both patients and HCWs at significant risk of acquiring occupational infections. Health authorities in the study area need to improve the training of HCWs and provision of infection prevention equipment. In addition, regular reporting, follow up and assessment of occupational exposures need to be introduced.

## Supporting Information

File S1 Questionnaire
(0.16 MB DOC)
