# Supplementary material for: Standard Precautions: Occupational Exposure and Behavior of Health Care Workers in Ethiopia
Source: PLoS One. 2010 Dec 23;5(12):e14420. doi: 10.1371/journal.pone.0014420 (PMC3009714; doi:10.1371/journal.pone.0014420)
Supplement: File S1 — Questionnaire (0.16 MB DOC) [file pone.0014420.s001.doc]

**Questionnaire**

A cross- sectional study on factors influencing occupational exposure of health care workers to HIV/AIDS in all health centers and hospitals found in Dire Dawa administrative council and Harari region, Ethiopia, 2010.

Standard precaution is a term that you will frequently encounter in this questionnaire. Standard precautions is defined as; an official Occupational Safety and Health Administration (OSHA) terminology which refers to mandatory precautions which apply when a contact with blood and other body fluids (with the exception of sweat) may occur. The precautions are to be used in the care of all clients and patients, regardless of diagnosis or infection status.

| **Code no. _______** Name of your institution ____________________________ **Date ___/___/___.** | | | | | | | |
| --- | --- | --- | --- | --- | --- | --- | --- |
| **Part I: General information:** | | | | | | | |
| S/N | Question | Response | | | Code | | Skip |
| 001 | Age | ( ) Years | | |  | |  |
| 002 | Sex | ( ) Male  ( ) Female | | | 1  2 | |  |
| 003 | Marital status | ( ) Single  ( ) Married  ( ) Divorced/separated  ( ) Widowed | | | 1  2  3  4 | |  |
| 004 | Level of your institution | ( ) Hospital  ( ) Health center  ( ) Other specifies | | | 1  2  3 | |  |
| 005 | What is your profession? | ( ) Physician  ( ) Nurse (All type of nurse)  ( ) Health assistant  ( ) Lab. Technician  ( ) Midwife  ( ) Others specify ______________ | | | 1  2  3  4  5  6 | |  |
| 006 | Years of experience | ( ) Years | | |  | |  |
| 007 | What is your position/job currently in the institution? | ( ________________________________ ). | | |  | |  |
| 008. | Do you have another responsibility other than your position? | ( ) Yes (If yes specify) _______________.  ( ) No, I don’t have | | | 1  2 | |  |
|  |  |  | | |  | |  |
| **Part II:** The following questions are introduced to assess the **perception and feeling** you have on working conditions in your **institution**. Please select one from the choice**.** | | | | | | | |
| S/N | Question | | Response | Code | | Skip | |
| 009 | Do you think that you regularly apply Universal/standard precaution in your institutional usual practice? | | ( ) Always  ( ) Usually  ( ) Sometimes  ( ) Seldom  ( ) Never | 1  2  3  4  5 | |  | |
| 010 | Do you think that you are working to reduce nosocomial infections in your institution? | | ( ) Always  ( ) Sometimes  ( ) Never | 1  2  3 | |  | |
| 011 | Do you think that you are secured from any infection? | | ( ) Always  ( ) Usually  ( ) Sometimes  ( ) Seldom  ( ) Never | 1  2  3  4  5 | |  | |
| 012 | Are you satisfied by training /education opportunity on standard precaution in the institution? | | ( ) Very satisfied  ( ) Satisfied  ( ) No difference  ( ) Dissatisfied  ( ) Very dissatisfied | 1  2  3  4  5 | |  | |
| 013 | Are you satisfied by the personal protective materials supplied by your institution for implementing standard precautions effectively? | | ( ) Very satisfied  ( ) Satisfied  ( ) No difference  ( ) Dissatisfied  ( ) Very dissatisfied | 1  2  3  4  5 | |  | |
| 014 | Does your immediate supervisor involve you in decision making regarding solving the problems occurred in your working area? | | ( ) Always  ( ) Usually  ( ) Sometimes  ( ) Seldom  ( ) Never | 1  2  3  4  5 | |  | |

**Part III: In the table** below possible reasons that influence occupational exposure are listed. Please tick the level of importance of the factors listed in the table.

| S.N | Possible factors that may influence occupational exposure | Very weak importance | Weak importance | Medium importance | Strong importance | Very strong importance |
| --- | --- | --- | --- | --- | --- | --- |
|  |  | 1 | 2 | 3 | 4 | 5 |
| 1 | Overcrowded work place (wards, outpatient department, lab etc) |  |  |  |  |  |
| 2 | Lack of health care workers and work load |  |  |  |  |  |
| 3 | Lack of personal protection equipment |  |  |  |  |  |
| 4 | Lack of commitment on the part of health facility to invest in infection control programs |  |  |  |  |  |
| 5 | Lack of guideline on standard precaution in the health facility |  |  |  |  |  |
| 6 | Lack of awareness about standard precautions in health care settings |  |  |  |  |  |
| 7 | Inadequate hand washing facility |  |  |  |  |  |

Part IV: Question on KAP of health care workers on Standard precautions and HIV/AIDS

| **SECTION I;** PRACTIES TO WARDS STANDARD PRECAUTIONS & HIV/AIDS  **(Should be answered by ticking in the space provided )** | | | | |
| --- | --- | --- | --- | --- |
| **S/ No** | **Question** | **Response** | **Code** | **Skip** |
| 101 | Do you practice standard blood and body fluid precautions at your workplace? | [ ] Always  [ ] Usually  [ ] Less frequently  [ ] Rarely  [ ] None at all  [ ] Don’t know | 1  2  3  4  5  6 |  |
| 102 | The last time you took a blood sample or contact with blood did you wear gloves? | [ ] Yes  [ ] No  [ ] Don’t know | 1  2  3 |  |
| 103 | Do you wash your hands before examining a patient? | [ ] Yes  [ ] No  [ ] Don’t know | 1  2  3 |  |
| 104 | Do you recap needles immediately after using them? | [ ] Yes  [ ] No  [ ] Don’t know | 1  2  3 |  |
| 105 | Do you treat blood spills on floors or other surfaces with a disinfectant before cleaning up? | [ ] Yes  [ ] No  [ ] Don’t know | 1  2  3 |  |
| 106 | Did you ever consider starting post exposure prophylaxes after a needle stick injury in your work place? | [ ] Yes  [ ] No  [ ] Don’t know | 1  2  3 |  |
| 107 | Do you wear personal protective equipments? | [ ] Yes  [ ] No | 1  2 | 109 |
| 108 | If yes to the above question (107), select from the list below? (It is possible to **circle** more than one item)  108.1 Apron  108.2 glove  108.3 Head cover  108.4 Boots/ shoe  108.5 Eye protectors / goggle  108.6 Mask  108.7 Examination glove  108.8 Gown  108.9 Other specify(----------------) | Yes No Don’t know  1 2 99  1 2 99  1 2 99  1 2 99  1 2 99  1 2 99  1 2 99  1 2 99  ( ) | |  |

| 109 | | | If your answer is No for question 107, Why?  109.1 Difficult to work with  109.2 Not always necessary  109.3 Uncomfortable  109.4 Unavailable’  109.5 Out of stock  109.6 Other specify______________ | Yes No Don’t know  1 2 99  1 2 99  1 2 99  1 2 99  1 2 99  ( ) | | | | | |  | |  | |
| --- | --- | --- | --- | --- | --- | --- | --- | --- | --- | --- | --- | --- | --- |
| 110 | | | How frequently do you wash your gown?  110.1 Three times per week  110.2 Two-times per week  110.3 Once per week  110.4 Once per two weeks  110.5 Other specify ________________ | Yes No Don’t know  1 2 99  1 2 99  1 2 99  1 2 99  ( ) | | | | | |  | |  | |
| 111 | | | Do you reuse syringe and needles? | [ ] Yes  [ ] No | | | 1  2 → | | | 113 | |  | |
| 112 | | | If you answered YES to the above question (111) What do you think are the main reason for reuse of syringe and needles? (It is possible to **circle** more than one item)  112.1 Shortage of supply  112.2 Knowledge deficit  112.3 Carelessness  112.4 to reduce the cost of treatment  112.5 Other specify _______________ | Yes NO Don’t know  1 2 99  1 2 99  1 2 99  1 2 99  ( ) | | | | | |  | |  | |
| 113 | | | Have you ever had needle stick injury? | [ ] Yes  [ ] No  [ ] Don’t know | | | | | | 1  2  99 | |  | |
| 114 | | Have you faced a needle sick injury in the last one year? | | [ ] Yes  [ ] No  [ ] Don’t know | | | | | 1  2 →  99 | | 116 | | |
| 115 | | If YES to question 115. How did you sustain the needle stick injury in the last one year? | | [ ] During recapping  [ ] By sudden movement of the patient  [ ] Other specify______ | | | | | 1  2  [ ] | |  | | |
| 116 | | Have you ever had sharp injury? | | [ ] Yes  [ ] No  [ ] Don’t know | | | | | 1  2  99 | |  | | |
| 117 | | Have you faced a sharp injury in the last one year? | | [ ] Yes  [ ] No  [ ] Don’t know | | | | | 1  2  99 | | 118 | | |
| 118 | | In your institution, is there any prophylaxis to HIV after injury by needle or sharps? | | [ ] Yes  [ ] No  [ ] Don’t know | | | | | 1  2  99 | |  | | |
| 119 | | Have you ever had blood or body fluid splash to your eye and/or mouth? | | [ ] Yes  [ ] No  [ ] Don’t know | | | | | 1  2  99 | |  | | |
| 120 | | Have you had blood or body fluid splash to your eye and/or mouth in the last one year? | | [ ] Yes  [ ] No  [ ] Don’t know | | | | | 1  2  99 | |  | | |
| 121 | | After being exposed to blood or body fluid through splashing to wet surfaces such as eyes or through sharps or needle stick injury, what measure (s) did you take? **Circle the number only if you have been exposed to blood or body fluids. Go to the next question if not exposed.**  121.1. Washing with soap and water  121.2. Wash with alcohol, iodine, chlorine  121.3. Applying pressure to stop bleeding  121.4. Dress the wound  121.5. Squeezing to extract more blood  121.6. Take TAT  121.7. Visiting VCT  121.8. Seek Post Exposure Prophylaxis  121.9. Report to the head person  121.10. other specify__________ | | Yes No Don’t know  1 2 99  1 2 99  1 2 99  1 2 99  1 2 99  1 2 99  1 2 99  1 2 99  1 2 99  ( ) | | | | | | |  | | |
|  | |  | |  | | | | | | |  | | |
|  | |  | |  | | | | | | |  | | |
| **SECTION II;** ATTITUDES TO STANDARD PRECAUTIONS & HIV/AIDS  (Place, tick in the space provided ) | | | | | | | | | | | | |  |
| S/ No | Question | | | | Response | Code | | Skip | | | | |  |
| 201 | When admitted to hospital, patients  who are HIV-positive should not be  put in rooms with other patients | | | | [ ] Strongly agree  [ ] Agree  [ ] Neutral  [ ] Disagree  [ ] Strongly disagree | 1  2  3  4  5 | |  | | | | |  |
| 202 | When caring for a person with HIV/  AIDS, you need to worry about  putting your family and friends at risk  of contracting the disease | | | | [ ] Strongly agree  [ ] Agree  [ ] Neutral  [ ] Disagree  [ ] Strongly disagree | 1  2  3  4  5 | |  | | | | |  |
| 203 | Healthcare workers in your institution worry  about getting HIV/AIDS while caring for patients | | | | [ ] Strongly agree  [ ] Agree  [ ] Neutral  [ ] Disagree  [ ] Strongly disagree | 1  2  3  4  5 | |  | | | | |  |
| 204 | Glove use for all patient care contacts is a useful strategy for reducing risk of transmission of organisms? | | | | [ ] Strongly agree  [ ] Agree  [ ] Neutral  [ ] Disagree  [ ] Strongly disagree | 1  2  3  4  5 | |  | | | | |  |
| 205 | In the absence of standard precaution health care facilities can be the source of infection and epidemic diseases? | | | | [ ] Strongly agree  [ ] Agree  [ ] Neutral  [ ] Disagree  [ ] Strongly disagree | 1  2  3  4  5 | |  | | | | |  |
| 206 | The risk of occupational HIV/AIDS infection among health workers in your work place is high | | | | [ ] Strongly agree  [ ] Agree  [ ] Neutral  [ ] Disagree  [ ] Strongly disagree | 1  2  3  4  5 | |  | | | | |  |

| **SECTION III;** KNOWLEDGE TO WARDS STANDARD PRECAUTION AND HIV/AIDS  (Place, tick in the space provided) | | | | |
| --- | --- | --- | --- | --- |
| S/ No | Question | Response | Code | Skip |
| 301 | Have you ever participated in any training programme about infection prevention or standard precaution? | [ ] Yes  [ ] No  [ ] Don’t Know | 1  2  99 |  |
| 302 | Which of the following disease can be transmitted through dirty needles and sharps?  302.1 Hepatitis (HBV)  302.2 Hepatitis (HCV)  302.3 HIV (AIDS)  302.4 Tetanus  302.5 Malaria  302.6 Tuberculosis  302.7 Other specify | Yes No Don’t know  1 2 99  1 2 99  1 2 99  1 2 99  1 2 99  1 2 99  ( ) | [ ]  [ ]  [ ]  [ ]  [ ]  [ ] |  |
| 303 | Do you think you are at risk of acquiring HIV in your workplace? | [ ] Yes  [ ] No  [ ] Don’t Know | 1  2  99 |  |
| 304 | Do you think your clients may have acquired HIV through the service they get in your health care facility? | [ ] Yes  [ ] No  [ ] Don’t Know | 1  2  99 |  |
| 305 | Do you feel that you over prescribe injection in  your health care institution | [ ] Yes  [ ] No  [ ] Don’t Know | 1  2  99 |  |
| 306 | Is it safe to use syringe between patients if the needle is changed? | [ ] Yes  [ ] No  [ ] Don’t Know | 1  2  99 |  |

| S/ No | Question | Response | Code | Skip |
| --- | --- | --- | --- | --- |
| 307 | Where do you dispose sharp materials such as used needles?  307.1 Open pail  307.2 In sharp and liquid proof container with  out removing syringe  307.3 In sharp and liquid proof container after  separating the needle from the syringe  307.4 Mixed with other wastes/ rubbish  307.5 Other specify ( ) | Yes No Don’t Know  1 2 99  1 2 99  1 2 99  1 2 99  1 2 99 | [ ]  [ ]  [ ]  [ ]  [ ] |  |
| 308 | Gloves and gowns are required for any contact with patients | [ ] Yes  [ ] No  [ ] Don’t Know | 1  2  99 |  |
| 309 | To prevent accidental injury, contaminated needles should be recapped immediately after use | [ ] Yes  [ ] No  [ ] Don’t Know | 1  2  99 |  |

| **Thank you very much**  for participating in this study.  The information you have provided will contribute to understanding the factors which influence compliance to standard precautions. |
| --- |
